# 713 Prevalence and Outcomes of Toxin Producing Staphylococcus Aureus in Paediatric Burn Wounds

**DOI:** 10.1093/jbcr/irae036.257

**Published:** 2024-04-17

**Authors:** Adam Couves, David McGill, Sharon Ramsay, Nikolaos Arkoulis

**Affiliations:** Glasgow Royal Infirmary, Glasgow, Scotland; Greater Glasgow and Clyde, Glasgow, Scotland; RHSC, Glasgow, Scotland; GRI, Glasgow, Scotland; Glasgow Royal Infirmary, Glasgow, Scotland; Greater Glasgow and Clyde, Glasgow, Scotland; RHSC, Glasgow, Scotland; GRI, Glasgow, Scotland; Glasgow Royal Infirmary, Glasgow, Scotland; Greater Glasgow and Clyde, Glasgow, Scotland; RHSC, Glasgow, Scotland; GRI, Glasgow, Scotland; Glasgow Royal Infirmary, Glasgow, Scotland; Greater Glasgow and Clyde, Glasgow, Scotland; RHSC, Glasgow, Scotland; GRI, Glasgow, Scotland

## Abstract

**Introduction:**

Toxic Shock Syndrome is a rare but serious complication of burn injury in children. Illness is mediated by Toxic Shock Syndrome Toxin -1 (TSST-1) producing strains of staphylococcus aureus. Little is known about the prevalence of TSST-1 producing staphylococcus aureus in burn wounds and as such the role of antibiotic prophylaxis is unclear.

**Methods:**

A prospectively maintained buns database was retrospectively reviewed for all cases of burn injury in paediatric patients of any aetiology over a three year period. Patients presented as inpatients or outpatients to the national burns centre in Scotland, UK. Patients were aged 0-16 years. Burn wounds were routinely swabbed on initial review. Specimens yielding staphylococcus aureus were sent for an overnight culture in enrichment medium followed by a commercially available latex agglutination assay to determine TSST-1 production. Electronic patient notes were reviewed to correlate clinical outcomes in these cases.

**Results:**

1313 children attended with burn injuries with a total of 1365 swab results. No bacterial growth was present in most cultures (69%). The most common pathogen yielded was staphylococcus aureus: 293 results (21%) of which 12 (0.8%) were TSST-1 producing. All TSST-1 strains were sensitive to flucloxacillin. 3 patients (all under 2 years old) developed signs of toxic shock syndrome: 1 was already an inpatient and 2 were admitted from the community who had previously not been referred to the burns service and had not received standard treatment. No patients had severely altered physiology to warrant admission to a critical care environment or require anti-toxin therapy with fresh frozen plasma or immunoglobulin.

**Conclusions:**

4% of staphylococcus aureus strains in our patient population were TSST-1 producing under laboratory conditions. The prevalence of TSST-1 producing staphylococcus aureus in paediatric burn wounds is 0.9%. We have demonstrated an acceptable safety profile of not giving routine antibiotic prophylaxis in paediatric burns to prevent toxic shock syndrome.

**Applicability of Research to Practice:**

Latex agglutination assays to determine toxin production is an effective way of highlighting potentially at-risk patients to clinicians to refine the population exposed to prophylactic antibiotics.

